# Extraocular muscle enlargement

**DOI:** 10.1007/s00417-022-05727-1

**Published:** 2022-06-17

**Authors:** Khizar Rana, Valerie Juniat, Sandy Patel, Dinesh Selva

**Affiliations:** 1 Department of Ophthalmology & Visual Sciences, University of Adelaide, North Terrace, Adelaide, SA 5000 Australia; 2 South Australian Institute of Ophthalmology, Royal Adelaide Hospital, Port Road, Adelaide, SA 5000 Australia; 3 Department of Medical Imaging, Royal Adelaide Hospital, Port Road, Adelaide, SA 5000 Australia

**Keywords:** Extraocular muscle, Computed tomography, Magnetic resonance imaging, Orbit

## Abstract

Extraocular muscle enlargement can occur secondary to a range of orbital and systemic diseases. Although the most common cause of extraocular muscle enlargement is thyroid eye disease, a range of other inflammatory, infective, neoplastic, and vascular conditions can alter the size and shape of the extraocular muscles. Imaging with computed tomography and magnetic resonance imaging plays an essential role in the workup of these conditions. This article provides an image-rich review of the wide range of pathology that can cause enlargement of the extraocular muscles.


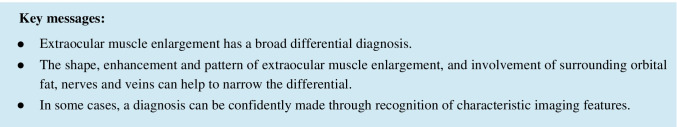


## Introduction

Extraocular muscle enlargement (EOME) can occur secondary to an array of inflammatory, neoplastic, infective or vascular conditions. Orbital imaging with computed tomography (CT) or magnetic resonance imaging (MRI) plays an essential role in the workup of these conditions. High-resolution orbital MRI has allowed for a more detailed characterisation of orbital disease. Recognising the pattern of muscle involvement along with accompanying orbital signs can help to narrow the differential diagnosis, or even enable a single diagnosis. This review focuses on the radiological findings associated with pathology that cause extraocular muscle enlargement.

### Thyroid eye disease

**Fig. 1:**
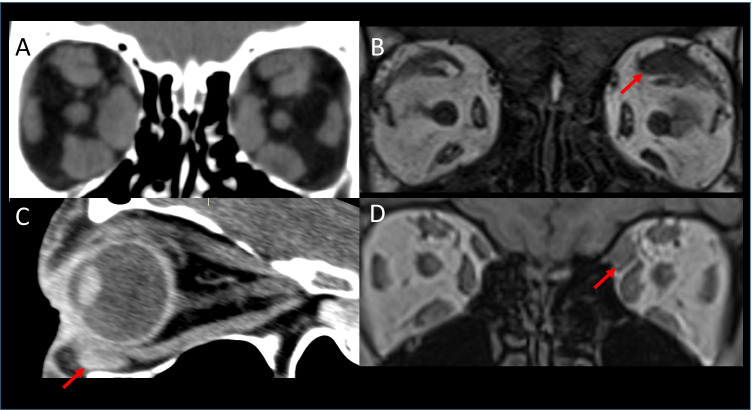
Thyroid eye disease. Coronal CT (**A**) of the orbit demonstrating a ‘typical’ case of TED with bilateral extraocular muscle enlargement of the inferior rectus, medial rectus and superior muscle group. Coronal T1-weighted MRI (**B**) showing enlargement of the left levator palpebrae superioris (arrow) without involvement of the other muscles. Quasi-sagittal CT (**C**) of the right orbit showing enlargement of the inferior oblique muscle (arrow) with relative sparing of the inferior rectus. T1-weighted coronal MRI (**D**) showing predominant enlargement of the left superior oblique muscle (arrow)

Thyroid eye disease (TED) is the most common inflammatory myopathy affecting the extraocular muscles. Radiologically, it presents with bilateral enlargement of the muscle belly with relative sparing of the anterior tendon. Traditionally, muscles are said to be involved in the following order: inferior rectus, medial rectus, superior rectus and lateral rectus (Fig. 1A) [1]. However, it is now being recognised that superior muscle complex involvement is also very frequent, possibly the most common [2, 3]. Furthermore, isolated levator muscle enlargement can also occur (Fig. 1B) and is associated with upper eyelid retraction [2–4]. Both superior and inferior oblique involvement has also been recognised and should be assessed on imaging (Fig. 1C, D) [5, 6].

Most patients with TED have muscle enlargement. However, up to one-fourth of patients will have no fat or muscle enlargement, and a smaller proportion will only have fat expansion without muscle enlargement [7]. TED can also be accompanied by bilateral, unilateral or asymmetric lacrimal gland enlargement [8, 9].

TED is a clinical diagnosis. Imaging can assist in differentiating TED from alternative pathologies in unclear cases, identify active orbital inflammation, predict response to therapy and assist in diagnosing dysthyroid optic neuropathy.

### Dysthyroid optic neuropathy

Dysthyroid optic neuropathy (DON) is a sight-threatening complication of TED. A number of radiological features can assist in diagnosing DON: orbital apex crowding [10], higher extraocular muscle volumes [11], superior ophthalmic vein enlargement [12], effacement of the fat plane surrounding the optic nerve [13], anterior displacement of the lacrimal gland [13], enlarged tendons [14] and superior orbital fissure fat prolapse [15]. It should be noted that DON is essentially a clinical diagnosis of exclusion and absence of the aforementioned radiological signs does not preclude the diagnosis.

**Fig. 2:**
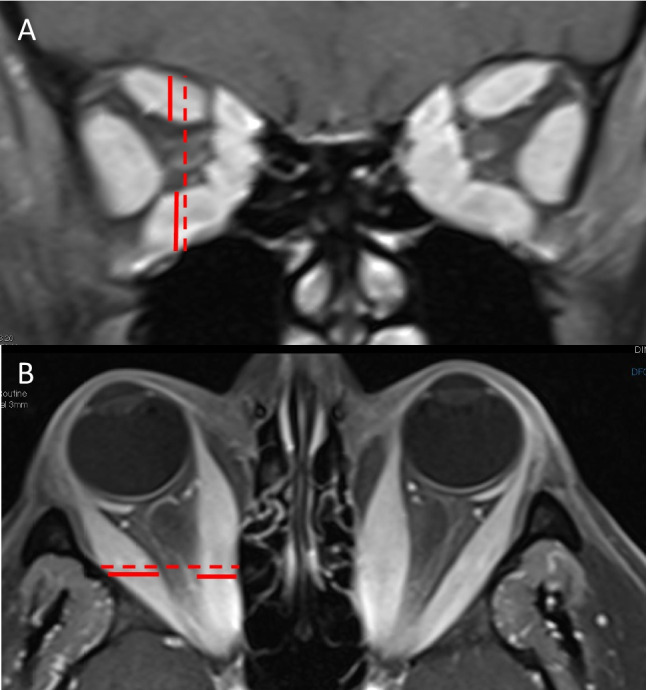
Right dysthyroid optic neuropathy. Mid-coronal fat-suppressed contrast-enhanced T1-weighted MRI (**A**) showing the vertical muscle index, which is a ratio of the sum of the vertical height of the superior and inferior recti (solid line) to the vertical orbital height (dashed line). Axial fat-suppressed contrast-enhanced T1-weighted MRI (**B**) showing the horizontal muscle index, which is a ratio of the sum of the diameters of the medial and lateral recti (solid line) to the horizontal orbital distance (dashed line)

A muscle index of greater than 50%, defined as the percentage of orbital height occupied by the superior and inferior recti or percentage of width occupied by the horizontal recti at the midpoint between the posterior globe and orbital apex, has a high sensitivity for DON (Fig. 2) [15, 16]. The anatomy of the bony orbit can also predispose patients to the development of DON. Patients with DON have shown to have narrower bony orbital angles than patients without DON for an identical muscle diameter index [10].

Enlargement of the superior muscle complex plays an important role in the development of DON [17, 18]. This correlates clinically with the inferior visual field deficits seen in TED. The ratio of the superior rectus-levator complex volume to the summated soft tissue volume is significantly higher in patients with DON, whereas no significant differences have been observed for the other extraocular muscles [18]. Anatomically, this may be explained by the fact that the superior rectus narrows vertically less than the inferior rectus as it approaches the annulus of Zinn.

### Active thyroid eye disease

**Fig. 3:**
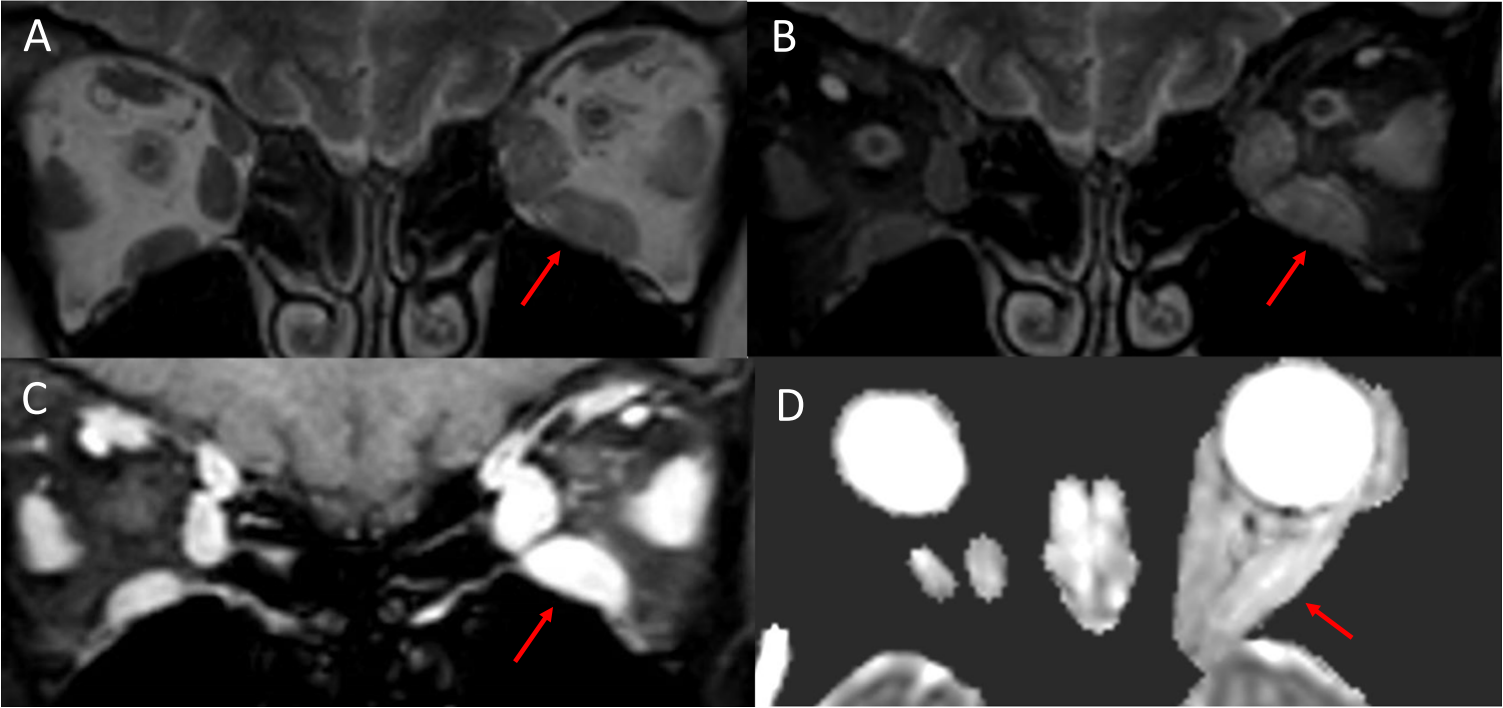
Active thyroid eye disease. Coronal T2-weighted MRI (**A**) showing enlargement and hyperintensity within the left inferior rectus, medial rectus and lateral rectus muscles (arrow). Coronal fat-suppressed T2-weighted MRI (**B**) demonstrating enlargement and hyperintensity of the left inferior rectus, medial rectus and lateral rectus (arrow). Coronal fat-suppressed T1-weighted MRI (**C**) showing relatively increased enhancement of the affected left extraocular muscles (arrow). ADC map (**D**) demonstrating a hyperintense signal within the left orbit (arrow)

**Fig. 4:**
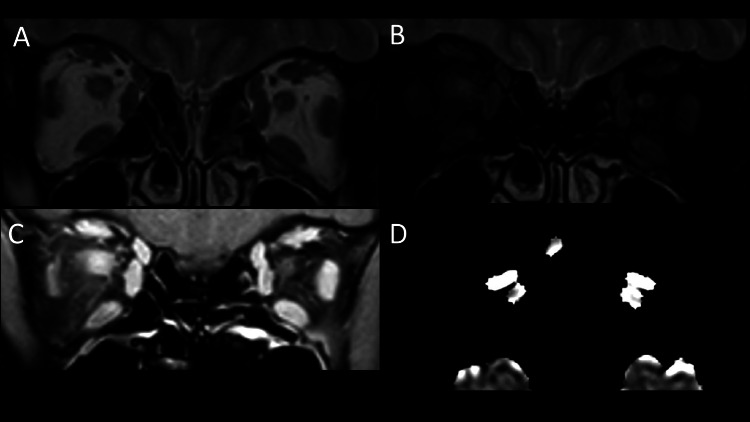
Normal extraocular muscles. Coronal T2-weighted MRI (**A**) demonstrating no muscle enlargement and no hyperintensity within the muscles.Coronal fat-suppressed T2-weighted MRI (**B**) showing symmetrical signal intensity of the muscles. Fat-suppressed contrast-enhanced T1-weighted MRI (**C**) showing symmetrical enhancement of the extraocular muscles. ADC map (**D**) showing no hyperintense signals within either orbit

Various imaging techniques have been used to assess orbital inflammation to help diagnose active disease and predict response to therapy. Active TED involves inflammatory oedema of the extraocular muscles which corresponds to T2-hyperintensity and increased contrast enhancement relative to unaffected extraocular muscles (Fig. 3) [19, 20]. This is in comparison to the findings seen in a normal patient (Fig. 4) with no T2-hyperintensity of the extraocular muscles, no significant or asymmetric extraocular muscle enhancement and no hyperintense signals on ADC map.

#### Signal intensity ratio

**Fig. 5:**
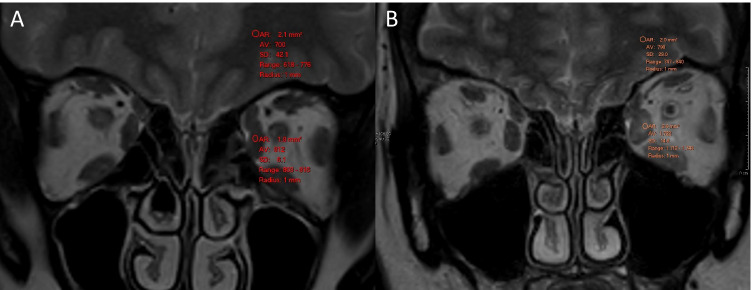
Signal Intensity ratio. Normal coronal T2-weighted MRI (A) showing the signal intensity ratio of the extraocular muscle and brain white mater. Coronal T2-weighted MRI (B) in a patient with active TED involving the left inferior and medial rectus muscles showing the signal intensity ratio between the inflamed left medial rectus and brain white mater

A higher signal intensity ratio (SIR), the ratio of signal intensity from the extraocular muscles to the signal intensity of normal tissue (e.g. brain white matter), is a marker of active TED and has shown correlation with the clinical activity score [21–23]. The SIR can be measured on STIR and T2-weighted sequences (Fig. 5) [19, 21–24]. The SIR usually decreases following immunosuppressive therapy or radiotherapy [25, 26]. Patients with persistently high SIRs despite medical therapy may be at an increased risk of further clinical deterioration [25].

#### Apparent diffusion coefficient

Diffusion-weighted imaging and the corresponding apparent diffusion coefficient (ADC) values can quantitatively and qualitatively assess the diffusion of water at a cellular level. Higher ADC values are seen in the acute inflammatory phase of TED and can help differentiate the active phase of TED from the inactive fibrotic phase (Fig. 3D) [27]. Hyperintense ADC signal within the extraocular muscles may be seen prior to any abnormality being seen on conventional MRI [28]. This may allow for earlier initiation of medical therapies in these patients. Additionally, ADC values can also help to monitor treatment response as they have shown correlation with the CAS and decrease following steroid therapy [27].

#### T2 relaxation time

T2 relaxation time (T2RT) is a tissue-specific time constant describing the decay of transverse magnetisation of tissues. Accordingly, changes within the cellular architecture of extraocular muscles can be quantitatively assessed with T2RTs. Patients with active TED have higher T2RT than inactive TED or healthy controls, and the T2RT has been correlated with the CAS and muscle areas [29–31]. A higher pre-intervention T2RT may be used to predict patients who are less likely to response to steroid therapy [32]. T2RT decreases following steroid therapy and can also be used to monitor the patient’s response to steroid therapy [30, 33].

### Inactive thyroid eye disease

Inactive TED is characterised by interstitial fibrosis, collagen deposition and fatty infiltration. Fatty infiltration can be seen as areas of hypodensity within the extraocular muscles on computed tomography and is significantly more common in patients with TED as compared to healthy controls [34]. Heterogenous areas of low signal intensity within the extraocular muscles on T2-weighted imaging may represent areas of fibrotic change and is more common in patients with irreversible diplopia following medical therapy [24].

#### Other inflammatory conditions

Myositis of the extraocular muscles can be secondary to idiopathic orbital myositis or a range of specific autoimmune conditions. McNab [35] has proposed a comprehensive classification system for orbital myositis focused on defining the underlying aetiology of the myositis.

### Idiopathic orbital myositis

**Fig. 6:**
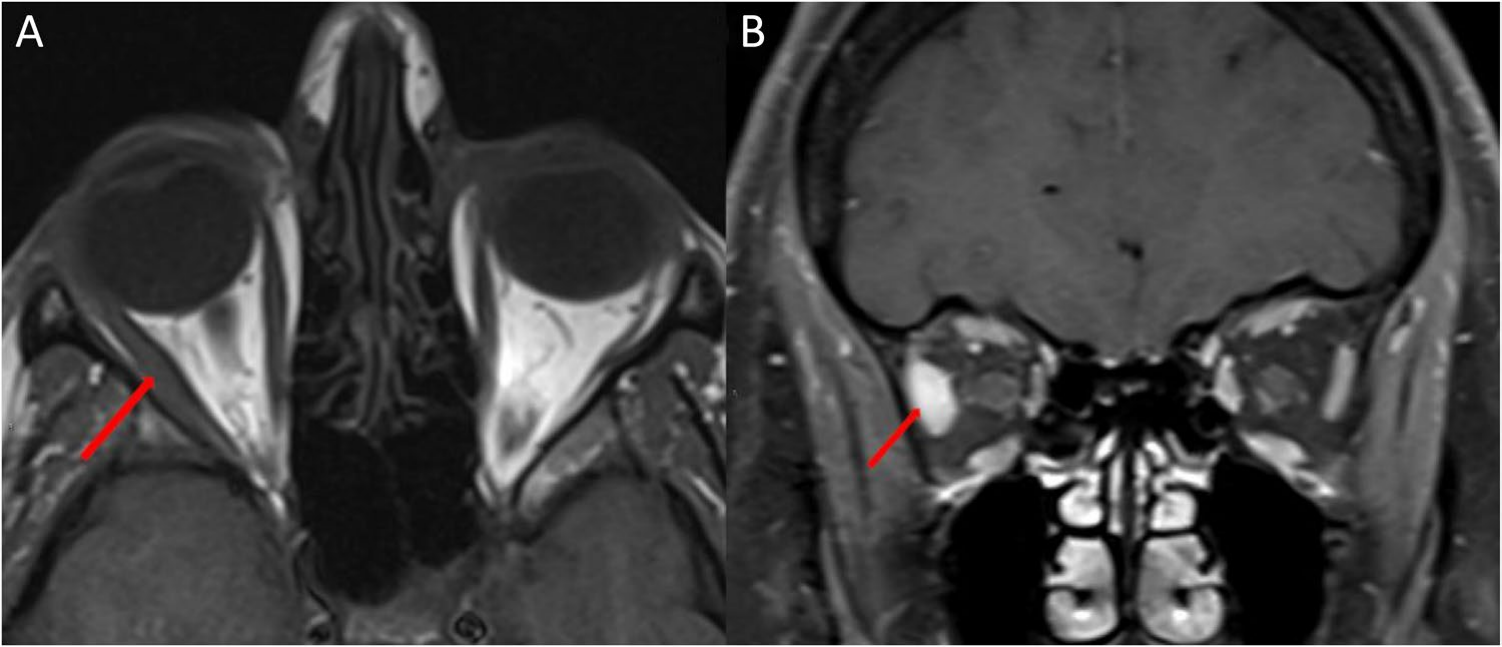
Idiopathic orbital myositis. Axial T1-weighted MRI (**A**) showing isointense enlargement of the right lateral rectus muscle including the anterior tendon (arrow). Coronal fat-suppressed contrast-enhanced T1-weighted MRI (**B**) showing enlargement and enhancement of the right lateral rectus muscle

Idiopathic orbital myositis is a subset of idiopathic orbital inflammatory syndrome or orbital pseudotumor. Idiopathic orbital myositis commonly presents unilaterally with single muscle involvement (Fig. 6) [36–38]. The medial or lateral recti are most commonly involved [39, 40]. Enlargement of the anterior muscle tendon can help differentiate it from TED; however, this is only seen in approximately half of all cases and in approximately 6% of TED patients [37, 38, 41, 42]. Orbital fat infiltration and lacrimal gland involvement can also be observed [43–45]. Multiple and bilateral muscle involvement and muscle tendon sparing are risk factors for recurrent disease [38, 46]. On MRI, the affected muscles are isointense on T1 and hyperintense on T2-weighted imaging [47–49].

#### Specific myositis

##### ***IgG4-related disease***

**Fig. 7:**
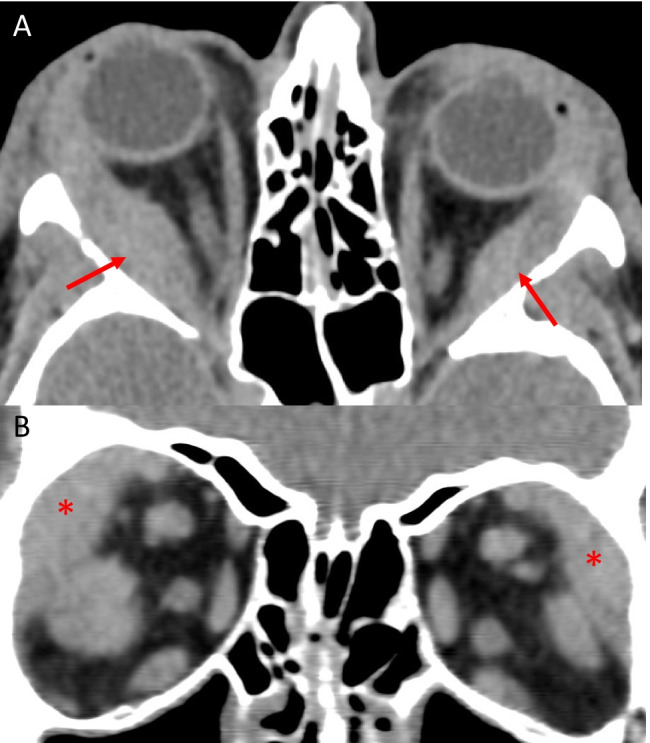
IgG4-related ophthalmic disease. Axial CT (**A**) showing bilateral enlargement of the lateral recti (R > L) including the anterior tendon (arrows). Coronal CT (**B**) demonstrating bilateral enlargement of the lacrimal glands (*)

IgG4-related disease (IgG4-RD) is a multisystemic inflammatory disease of unknown aetiology. IgG4-related ophthalmic disease (IgG4-ROD) is being increasingly recognised as a cause of previously labelled idiopathic orbital inflammation [50]. Bilateral enlargement of multiple muscles, particular the lateral rectus, is common (Fig. 7). This is usually accompanied by a combination of lacrimal gland enlargement, infraorbital or frontal nerve enlargement, sinus mucosal thickening, and pterygopalatine fossa involvement [51–54]. Trigeminal pathway involvement is a useful diagnostic sign for IgG4-ROD with one study finding that 50% of patients with infraorbital nerve enlargement had IgG4-ROD [55]. On CT, the muscles are isodense compared to unaffected muscles and on MRI muscles are isointense on T1 and hypoisointense lesions on T2 imaging are seen [53, 54, 56].

### Inflammatory bowel disease

Inflammatory bowel disease (IBD) consisting of ulcerative colitis and Crohn’s disease has been associated with orbital myositis. Presentation can be unilateral or bilateral with the medial and inferior recti most commonly involved [57–59]. Compared to idiopathic orbital myositis, bilateral muscle involvement is more common in the IBD group [35]. Fusiform enlargement of muscles with tendon sparing can mimic the presentation of TED [57, 59, 60]. The past medical history, thyroid hormone, and antibody studies can help make the distinction in such cases. The muscles can be large enough to cause optic nerve compression [57]. On MRI, the muscles are isointense on T1 and hyperintense on T2-weighted imaging [58, 60, 61].

### Sarcoidosis

The most common ocular presentation of sarcoidosis is with anterior uveitis with orbital myositis being relatively infrequent [62]. When affecting the muscles, bilateral disease with multiple muscle involvement and anterior tendon involvement is commonly seen [36, 63–66]. Involvement of surrounding structures can include the lacrimal gland, lateral wall of the cavernous sinus, and thickening and encasement of optic nerve producing a ‘tram-track’ sign [64, 67, 68]. The affected muscles are isointense on T1 and hypo-isointense on T2 with potential areas of higher intensity nodules [66–68].

### Granulomatosis with polyangiitis

**Fig. 8:**
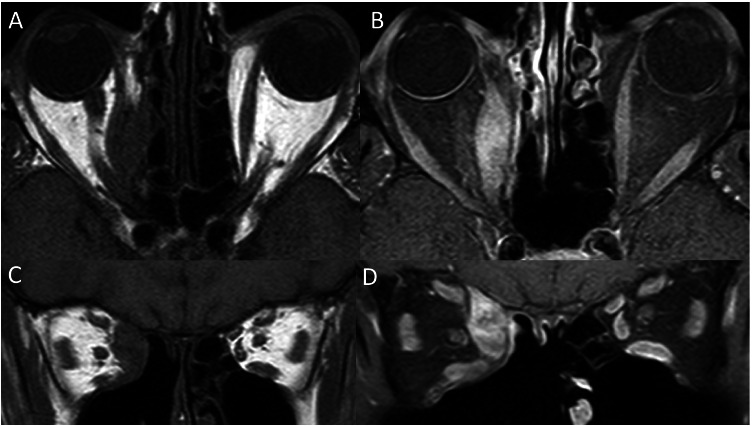
Granulomatosis with polyangiitis (Wegener’s). Axial T1-weighted MRI (**A**) and fat-suppressed contrast-enhanced T1-weighted MRI (B) showing fusiform enlargement of the right medial rectus muscle with anterior tendon sparing. Coronal T1-weighted MRI (**C**) demonstrates isointense enlargement of the right medial rectus. Coronal fat-suppressed contrast-enhanced T1-weighted MRI (**D**) shows heterogenous enhancement and enlargement of the right medial rectus

Granulomatosis with polyangiitis (GPA) is a multisystemic autoimmune disorder characterised by necrotising granulomatous inflammation. Orbital involvement occurs in up to 50% of GPA patients usually presenting with an orbital mass, or dacryoadenitis with isolated extraocular myositis being uncommon [69]. Concomitant sinus and nasal disease is common. Pachymeningeal enhancement may occur [70]. Extraocular myositis usually presents unilaterally with fusiform muscle enlargement and tendon sparing (Fig. 8) [36, 71–73]. Unilateral or bilateral involvement is possible [74–76]. The affected muscles are isointense on T1 [72]. Areas of hypoenhancement following contrast administration representing zonal necrosis may be suggestive of GPA [73]. On T2, a high signal intensity with a rim of hypointensity may also represent intralesional necrosis suggestive of GPA [71].

A range of other autoimmune conditions can be associated with orbital myositis including systemic/discoid lupus erythematosus [77, 78], giant-cell myositis [79–82], rheumatoid arthritis [83] and psoriasis [84]. Orbital myositis can occur secondary to drug reactions from the newer monoclonal antibodies, bisphosphonates or statins [35]. The pattern of enlargement is similar to idiopathic orbital myositis, with the muscles showing T1 isointensity and T2 hyperintensity.

#### Neoplastic conditions

A number of tumours can metastasise to the orbit causing extraocular muscle enlargement. Uncommonly, EOME may be the initial manifestation of a previously undiagnosed tumour [85].

##### *Lymphoma*

**Fig. 9:**
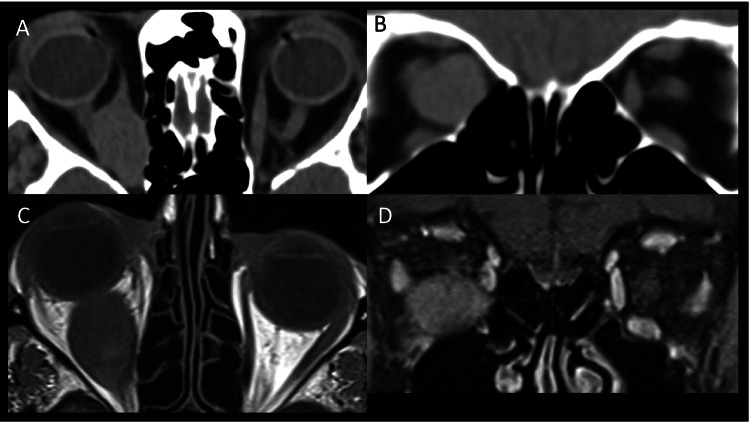
Intramuscular lymphoma. Case 1—axial (**A**) and coronal (**B**) CT scan showing significant enlargement of the right medial rectus muscle without involving the anterior tendon and causing optic nerve displacement. Case 2—axial T1-weighted MRI (**C**) shows isointense enlargement of the right inferior rectus muscle and right proptosis. Coronal fat-suppressed contrast-enhanced T1-weighted MRI (**D**) demonstrates heterogenous enlargement of the right inferior rectus muscle

Orbital lymphoma is the most common malignant tumour of the orbit in adults [86]. Intramuscular involvement is uncommon [87]. Extraocular muscle lymphoma presents unilaterally with either fusiform muscle enlargement and tendon sparing or diffuse muscle enlargement with tendon involvement (Fig. 9) [88–93]. Lymphoma is hypoisointense on T1 and iso to hyperintense on T2 imaging [75, 89, 94–96]. The presence of an orbital mass or lacrimal gland involvement that moulds to the globe or bone can help aid the diagnosis of lymphoma [89, 97–102].

##### *Breast cancer*

**Fig. 10:**
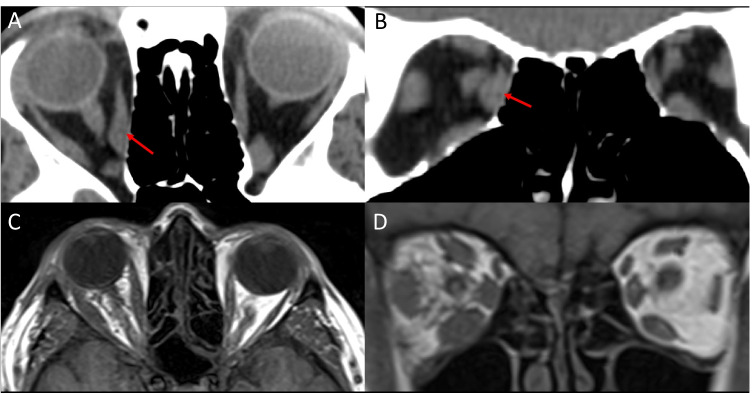
Breast cancer metastasis. Case 1—axial CT image of the orbits (**A**) demonstrating focal enlargement of the right medial medial rectus (arrow). Coronal CT image (**B**) demonstrates enlargement of the right medial rectus which is approaching the optic nerve. Case 2—axial T1-weighted MRI (**C**) demonstrating isointense enlargement of the medial and lateral recti muscles and intraconal fat infiltration. Coronal T1-weighted MRI (**D**) shows diffuse enlargement of the right extraocular muscles with intraconal fat infiltration

Breast cancer is one of the most common primary tumours to metastasise to the orbit [75, 103]. Breast cancer may present with unilateral [95, 104, 105] or bilateral extraocular muscle involvement (Fig. 10A, B) [106–108]. The horizontal recti are most commonly involved; however, involvement of the obliques has also been reported [85, 105, 108]. It commonly presents with fusiform muscle enlargement of affected muscles with tendon sparing [85, 108–112]. On MRI, the lesion is iso-hyperintense on T1- and T2-weighted imaging (Fig. 10C, D) [95, 104, 110, 113] with homogenous contrast enhancement [105, 112, 114].

##### *Melanoma*

**Fig. 11:**
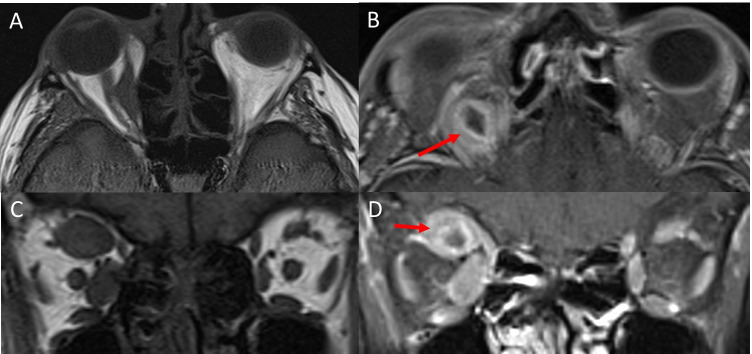
Melanoma metastasis. An axial T1-weighted MRI (**A**) shows isointense nodular enlargement of the right medial rectus muscle without involvement of the anterior tendon. An axial, contrast-enhanced, fat-supressed T1-weighted image (**B**) through the superior orbit shows an area of central hypointensity (arrow) with increased peripheral rim enhancement. Coronal T1-weighted MRI (**C**) shows isointense enlargement of the right medial rectus and superior rectus muscles. A coronal, contrast-enhanced, fat-supressed T1-weighted image (**D**) demonstrates enlargement and enhancement of the right medial and superior rectus muscles, with a central area of hypoenhancement in the right superior rectus (arrow)

Melanoma most commonly involves the medial rectus muscle [95, 115–117]. Well-defined focal nodular enlargement of muscles without involvement of the anterior tendon is most common [115, 116, 118]. On CT, the affected muscles are isodense compared to unaffected muscles. On MRI, the lesion is iso-hyperintense on T1 and hypo-isointense on T2 imaging. Heterogenous contrast enhancement with areas of central hypointensity reflecting haemorrhage, and increased peripheral rim enhancement can be seen (Fig. 11) [111, 115, 119]. Atypical presentations showing cystic masses with fluid–fluid levels have also been reported [118].

##### *Neuroendocrine tumour*

**Fig. 12:**
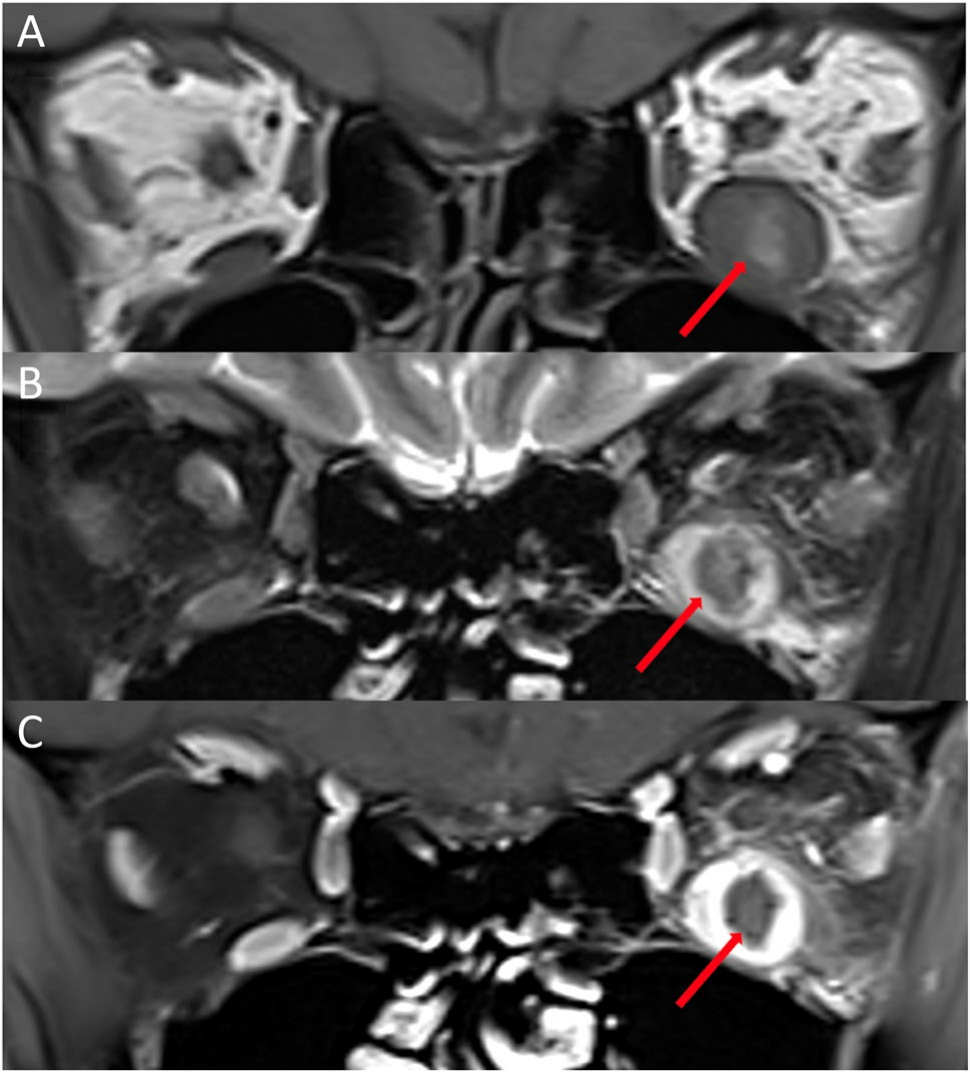
Neuroendocrine tumour metastasis. Coronal T1-weighted MRI (**A**), fat-suppressed T2-weighted MRI (**B**), and fat-suppressed contrast-enhanced T1-weighted MRI (**C**) showing enlargement of the left inferior rectus muscle with intralesional haemorrhage (arrows) suggested by the hyperintensity on T1-weighted scan (**A**) and hypointensity on fat-suppressed T2-weighted (**B**) and fat-suppressed T1-weighted contrast-enhanced scans (**C**)

Neuroendocrine tumours originate from the enterochromaffin cells of the gastrointestinal tract or bronchial lining. Unilateral presentation is most common with involvement of any of the extraocular muscles including the obliques [120–122]. Well-defined focal intramuscular masses are seen on imaging [95, 120, 122–125]. The masses may be of a significant size causing compression of adjacent structures including the optic nerve [95, 124–126]. They are isointense on T1 and hypo-isointense on T2-weighted imaging [95, 123, 127]. Heterogenous contrast enhancement can be seen [122, 124, 128]. Intralesional haemorrhage may be seen as ovoid areas of low signal intensity following contrast administration (Fig. 12) [129, 130].

##### *Gastric adenocarcinoma*

Gastric adenocarcinoma presents with diffuse muscle enlargement [117, 131, 132]. Nodular and fusiform enlargement of muscles has also been reported [133, 134]. The diffuse enlargement may involve the anterior muscle tendon [132]. Gastric adenocarcinoma appears isointense on T1 and iso-hyperintense on T2-weighted imaging [131, 134].

### Amyloidosis

**Fig. 13:**
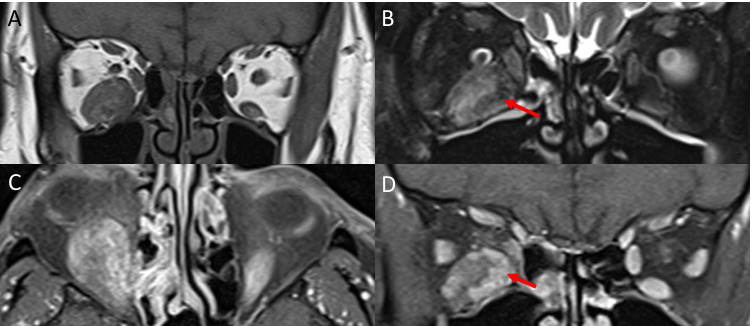
Intramuscular amyloidosis. Coronal T1-weighted MRI (**A**) demonstrating enlargement of the right inferior rectus muscle with areas of relative hyperintensity. Coronal T2-fat-suppressed MRI (**B**) shows enlargement and hypointense lesions of the right inferior rectus muscle (arrow). An axial fat-suppressed contrast-enhanced T1-weighted MRI (**C**) shows heterogenous pattern of enhancement of the right inferior rectus. Coronal fat-suppressed contrast-enhanced T1-weighted MRI (**D**) demonstrated a heterogenous pattern of enhancement, with areas of increased enhancement corresponding to T2-hypointense areas (arrow)

Amyloidosis is a multisystemic disease with deposition of amyloid protein occurring in various parts of the body. Amyloidosis can uncommonly infiltrate the extraocular muscles presenting with unilateral or bilateral involvement of the extraocular muscles [135–137]. The horizontal recti are most commonly involved [138]. It usually causes tendon sparing fusiform enlargement of the affected muscles [138–142] and can mimic TED particularly when it involves the inferior or medial recti [142, 143]. Calcified lesions infiltrating the extraocular muscles can however be seen on CT helping to differentiate it from TED [139, 142]. Adjacent hyperostosis and bony irregularity can support a diagnosis of amyloidosis [144]. On T1-weighted imaging, the enlarged muscles are isointense to the extraocular muscles [137, 139, 141]. On T2-weighted imaging, hypointense lesions can be seen reflecting the amyloid involvement (Fig. 13) [135, 139]. Contrast-enhanced scans may reveal heterogenous enhancement with patchy areas of reduced enhancement [135, 140, 141].

### Vascular conditions

Vascular malformations can cause extraocular muscle enlargement. Arteriovenous malformations cause an increase in pressure within the cavernous sinus resulting in decreased venous outflow from the superior ophthalmic vein. The resultant elevated capillary pressure causes enlargement of the extraocular muscles.

Primary vascular malformations of the orbit can also lead to extraocular muscle enlargement by direct infiltration of the muscles, arteriovenous shunting or haemorrhage within the muscles. This is a separate section later anyway. Intraorbital arteriovenous malformations or AV malformations secondary to retinoencephalofacial angiomatosis or facial angiomatosis have also been reported to cause EOME [145–147].

#### Carotid-cavernous fistula

**Fig. 14:**
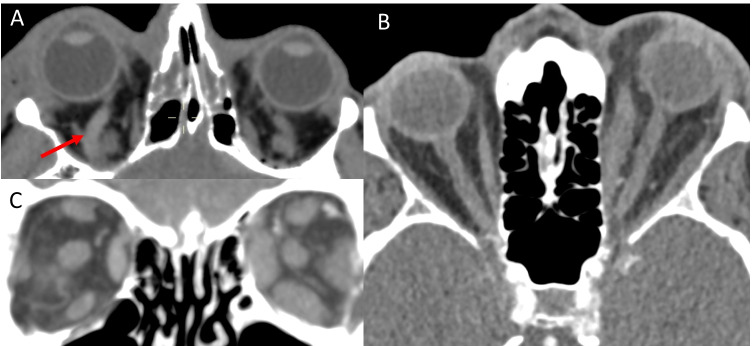
Carotid-cavernous fistula. Case 1—axial CT (**A**) of the orbits demonstrates enlargement of the right superior ophthalmic vein (arrow) and proptosis in a patient with a right direct carotid-cavernous fistula. Case 2—axial CT (**B**) showing left proptosis, enlargement of the left medial rectus muscle along with left globe tenting. Case 2—coronal CT (**C**) demonstrating enlargement of the left extraocular muscles

The description of the pattern of muscle enlargement in patients with carotid-cavernous fistulas (CCF) is limited in the literature. Accompanying radiological signs can often help aid the diagnosis. These signs include a dilatation of the superior ophthalmic vein which is seen in more than 80% of CCF cases (Fig. 14A) [46, 147, 148]. Other supportive findings include internal signal void within the cavernous sinus, and prominent venous drainage in the anterior, posterior or lateral venous sinuses [149]. Unilateral enlargement of multiple extraocular muscles can occur (Fig. 14B, C). Enlargement of the extraocular muscles will generally involve all the muscles on the affected side and may be accompanied by intraconal fat stranding [149]. Expansion and bulging of the lateral wall of the cavernous sinus is more prominent on the fistula side [148, 150]. If the intercommunicating vessels within the cavernous sinus are large enough, there may be bilateral expansion of the cavernous sinuses and involvement of both eyes [148].

#### Spontaneous haemorrhage related to the extraocular muscles

Nontraumatic orbital haemorrhage within the extraocular muscle belly or related to the muscle sheath can occur spontaneously in the absence of underlying vascular malformations. It usually presents in older females often with a history of hypertension and hyperlipidaemia [151]. Intra/juxtamuscular haemorrhage has a distinct radiological appearance which can help make the diagnosis and prevent unnecessary further investigations.

**Fig. 15:**
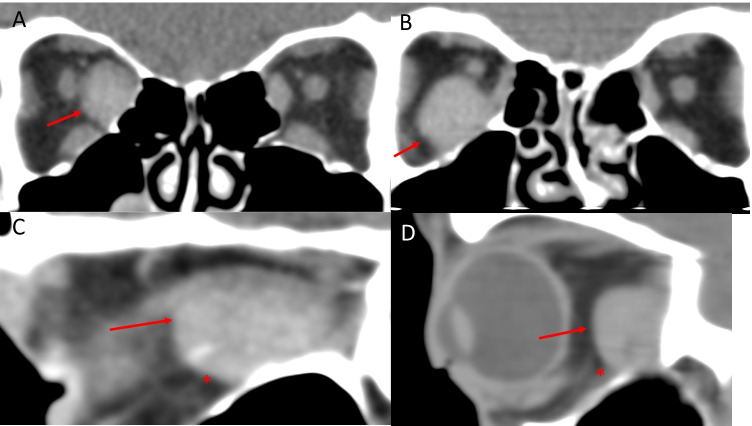
Two cases of Spontaneous haemorrhage related to extraocular muscles. Case 1—coronal (**A**) and sagittal (**C**) CT scans showing a large, well-defined mass in the right medial rectus with an anterior rounded border (arrow) and posterior tapering edge (*) towards the orbital apex. The sagittal image has a ‘bleached whale’ appearance suggestive of intramuscular haemorrhage. Case 2—coronal (**B**) and sagittal (**D**) CT scans showing a large, well-defined mass in the right inferior rectus with an anterior rounded border (arrow) and posterior tapering edge (*) towards the orbital apex

Intramuscular haemorrhage most commonly presents with unilateral involvement of the inferior rectus muscle [151, 152]. A large, often well-defined mass with an anterior rounded border and posterior tapering edge towards the orbital apex (“tear drop” appearance on axial scans and “beached whale” appearance on sagittal when involving the inferior rectus) is characteristic (Fig. 15) [151]. On CT, the haematoma is hyperdense if acute and hypo-isodense if subacute [153]. A fluid level may be observed in the clot, further supporting a diagnosis of a haematoma [152, 154]. Haemorrhage on MRI has a variable appearance depending on the age of the blood, stages of the clot and MRI sequence used. A fresh clot with deoxyhaemoglobin (< 2 days) is isointense on T1 and hypointense on T2W-MRI [153]. As the clot changes to intracellular methemoglobin, the signal on T1W-MRI becomes hyperintense and hypointense on T2W-MRI [153]. Extracellular methemoglobin is hyperintense on T2 and is seen between 2 weeks and 2 months after the initial haemorrhage. Serial MRIs can help to identify these changes and support the diagnosis of an intramuscular haemorrhage. Although gradient sequences are distorted at the level of the orbit, some sequences such as the B_0_ of the diffusion sequence may demonstrate low signal in the acute phase (susceptibility artefact) suggesting haemorrhage.

### Infective conditions

#### Cysticercosis

**Fig. 16:**
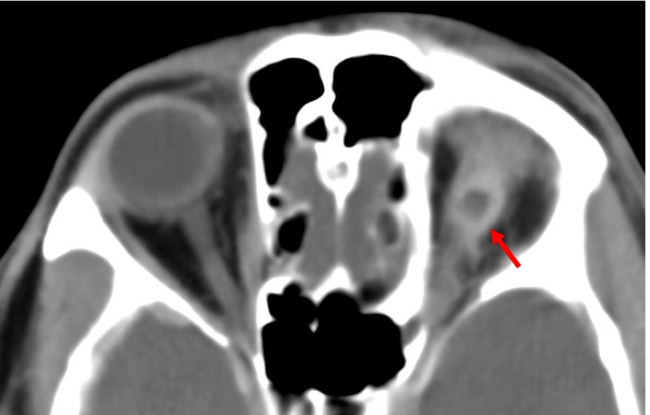
Cysticercosis. Contrast-enhanced CT of the orbits demonstrates a hypodense cystic mass within the left superior rectus with peripheral rim enhancement (arrow)

Cysticercosis is a parasitic infection caused by *Cysticercus cellulosae*, which is endemic in regions with poor sanitation. Orbital or extraocular muscle cysticercosis most commonly presents in teenagers [155, 156]. Extraocular muscle cysticercosis presents unilaterally with single muscle involvement of any of the extraocular muscles including the obliques [155]. Rath, Honavar, Naik, Anand, Agarwal, Krishnaiah and Sekhar [156] reviewed 138 cases of extraocular muscle cysticercosis and reported the superior rectus muscle to be most commonly involved, followed by the inferior, medial and lateral recti in descending order. On CT, imaging reveals an intramuscular hypodense, ring enhancing cystic lesion (Fig. 16), often in association with a scolex in approximately 50% of cases [156–158]. On T2-weighted imaging, a round hyperintense nodule can be seen on a hypointense background [159, 160] and on T1-fat-suppressed post contrast, a ring enhancing lesion with a hypointense central nodule is typical [161, 162].

#### Hydatid cyst

Hydatid cysts result from infection with the parasite *Echinococus granulosus* and can result in cyst formation anywhere in the body. Extraocular muscle hydatid cysts are rare. On MRI, it presents as a multiloculated cystic lesion which is hypointense on T1 and demonstrates peripheral rim enhancement following contrast administration [163].

#### Pyomyositis

Pyomyositis is an acute bacterial infection of skeletal muscle, usually caused by *Staphylococcus aureus*. It has a characteristic appearance on imaging. It presents unilaterally with involvement of any of the extraocular muscles. On CT, a round-oval hypodense lesion with rim enhancement is characteristically observed [164–166]. On T1-weighted imaging, a hypointense central lesion with rim enhancement can be seen, alongside oedema and enlargement of the affected muscle [166–168]. An abscess within the muscle usually results in restricted diffusion.

#### Orbital cellulitis

**Fig. 17:**
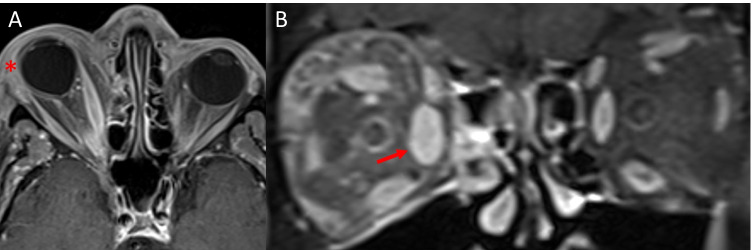
Orbital cellulitis. Axial fat-suppressed contrast-enhanced T1-weighted MRI (**A**) demonstrating preseptal soft tissue swelling (asterisks, *), enlargement and enhancement of the right medial rectus muscle and right axial proptosis. Coronal fat-suppressed contrast-enhanced T1-weighted MRI (**B**) demonstrates enlargement of the right medial rectus muscle (arrow) with associated intraconal fat stranding

Orbital cellulitis most commonly occurs from direct extension of adjacent sinus infection or preseptal cellulitis and imaging may reveal inflammation in these adjacent areas. Any of the extraocular muscles can be enlarged in orbital cellulitis (Fig. 17) [169]. Associated features include intraconal fat stranding, dacryoadenitis and optic perineuritis [170]. Potential complications include orbital abscess formation, intracranial extension and cavernous sinus thrombosis [170, 171].

## Conclusion

Extraocular muscle enlargement is most commonly due to thyroid eye disease; however, it can occur secondary to a number of other inflammatory, vascular or neoplastic conditions. Imaging is playing an increasingly important role in the characterisation of orbital diseases and can aid the diagnosis and management of orbital conditions. Ultimately, the diagnosis will be made in conjunction with an understanding of the patient’s demographics, past medical history and presentation. In cases where there is diagnostic uncertainty, an orbital biopsy may be required for definitive diagnosis.

## References

[1] Enzmann DR, Donaldson SS, Kriss JP (1979). Appearance of Graves' disease on orbital computed tomography. J Comput Assist Tomogr.

[2] Davies MJ, Dolman PJ (2017). Levator Muscle Enlargement in Thyroid Eye Disease-Related Upper Eyelid Retraction. Ophthalmic Plast Reconstr Surg.

[3] Wang Y, Mettu P, Broadbent T, Radke P, Firl K, Shepherd JB, Couch SM, Nguyen A, Henderson AD, McCulley T, McClelland CM, Mokhtarzadeh A, Lee JA, Garrity MS, Harrison AR (2020). Thyroid eye disease presenting with superior rectus/levator complex enlargement. Orbit.

[4] Kozaki A, Inoue R, Ito G, Mizuno K, Inoue T (2014). Features of untreated Graves' disease patients with bilateral upper eyelid retraction. Invest Ophthalmol Vis Sci.

[5] Thacker NM, Velez FG, Demer JL, Rosenbaum AL (2005). Superior oblique muscle involvement in thyroid ophthalmopathy. J aapos.

[6] Kakizaki H, Zako M, Iwaki M (2007). Thyroid-associated inferior oblique myopathy. Ophthalmology.

[7] Regensburg NI, Wiersinga WM, Berendschot TT, Potgieser P, Mourits MP (2011). Do subtypes of graves' orbitopathy exist?. Ophthalmology.

[8] Bingham CM, Harris MA, Realini T, Nguyen J, Hogg JP, Sivak-Callcott JA (2014). Calculated computed tomography volumes of lacrimal glands and comparison to clinical findings in patients with thyroid eye disease. Ophthalmic Plast Reconstr Surg.

[9] Harris MA, Realini T, Hogg JP, Sivak-Callcott JA (2012). CT dimensions of the lacrimal gland in Graves orbitopathy. Ophthalmic Plast Reconstr Surg.

[10] Chan LL, Tan HE, Fook-Chong S, Teo TH, Lim LH, Seah LL (2009). Graves ophthalmopathy the bony orbit in optic neuropathy, its apical angular capacity and impact on prediction of risk. AJNR Am J Neuroradiol.

[11] Gonçalves AC, Silva LN, Gebrim EM, Matayoshi S, Monteiro ML (2012). Predicting dysthyroid optic neuropathy using computed tomography volumetric analyses of orbital structures. Clinics (Sao Paulo).

[12] Lima Bda R, Perry JD (2013). Superior ophthalmic vein enlargement and increased muscle index in dysthyroid optic neuropathy. Ophthalmic Plast Reconstr Surg.

[13] Maj E, Cieszanowski A, Miskiewicz P, Trautsolt K, Bednarczuk T, Samsel A, Krzeski A, Rowinski O (2013). The value of MR imaging for the diagnosis of optic nerve neuropathy in patients with graves' disease. Neuroradiology.

[14] Weis E, Heran MK, Jhamb A, Chan AK, Chiu JP, Hurley MC, Rootman J (2011). Clinical and soft-tissue computed tomographic predictors of dysthyroid optic neuropathy: refinement of the constellation of findings at presentation. Arch Ophthalmol.

[15] Giaconi JA, Kazim M, Rho T, Pfaff C (2002). CT scan evidence of dysthyroid optic neuropathy. Ophthalmic Plast Reconstr Surg.

[16] So NM, Lam WW, Cheng G, Metreweli C, Lam D (2000). Assessment of optic nerve compression in Graves' ophthalmopathy The usefulness of a quick T1-weighted sequence. Acta Radiol.

[17] Starks VS, Reinshagen KL, Lee NG, Freitag SK (2020). Visual field and orbital computed tomography correlation in dysthyroid optic neuropathy due to thyroid eye disease. Orbit.

[18] Oropesa S, Dunbar KE, Godfrey KJ, Callahan AB, Campbell AA, Kazim M (2019). Predominant contribution of superior rectus-levator complex enlargement to optic neuropathy and inferior visual field defects in thyroid eye disease. Ophthalmic Plast Reconstr Surg.

[19] Liu X, Su Y, Jiang M, Fang S, Huang Y, Li Y, Zhong S, Wang Y, Zhang S, Wu Y, Sun J, Fan X, Zhou H (2021). Application of magnetic resonance imaging in the evaluation of disease activity in Graves' ophthalmopathy. Endocr Pract.

[20] Cakirer S, Cakirer D, Basak M, Durmaz S, Altuntas Y, Yigit U (2004). Evaluation of extraocular muscles in the edematous phase of Graves ophthalmopathy on contrast-enhanced fat-suppressed magnetic resonance imaging. J Comput Assist Tomogr.

[21] Higashiyama T, Iwasa M, Ohji M (2017). Quantitative analysis of inflammation in orbital fat of thyroid-associated ophthalmopathy using MRI signal intensity. Sci Rep.

[22] Mayer E, Herdman G, Burnett C, Kabala J, Goddard P, Potts MJ (2001). Serial STIR magnetic resonance imaging correlates with clinical score of activity in thyroid disease. Eye (Lond).

[23] Mayer EJ, Fox DL, Herdman G, Hsuan J, Kabala J, Goddard P, Potts MJ, Lee RW (2005). Signal intensity, clinical activity and cross-sectional areas on MRI scans in thyroid eye disease. Eur J Radiol.

[24] Yokoyama N, Nagataki S, Uetani M, Ashizawa K, Eguchi K (2002). Role of magnetic resonance imaging in the assessment of disease activity in thyroid-associated ophthalmopathy. Thyroid.

[25] Higashiyama T, Nishida Y, Morino K, Ugi S, Nishio Y, Maegawa H, Ohji M (2015). Use of MRI signal intensity of extraocular muscles to evaluate methylprednisolone pulse therapy in thyroid-associated ophthalmopathy. Jpn J Ophthalmol.

[26] Wu Y, Tong B, Luo Y, Xie G, Xiong W (2015). Effect of radiotherapy on moderate and severe thyroid associated ophthalmopathy: a double blind and self-controlled study. Int J Clin Exp Med.

[27] Feeney C, Lingam RK, Lee V, Rahman F, Nagendran S (2020) Non-EPI-DWI for detection, disease monitoring, and clinical decision-making in thyroid eye disease. AJNR Am J Neuroradiol 41:1466-1472. 10.3174/ajnr.A6664 10.3174/ajnr.A6664 PMC7658861 32796099

[28] Kilicarslan R, Alkan A, Ilhan MM, Yetis H, Aralasmak A, Tasan E (2015). Graves' ophthalmopathy: the role of diffusion-weighted imaging in detecting involvement of extraocular muscles in early period of disease. Br J Radiol.

[29] Chen W, Hu H, Chen HH, Su GY, Yang T, Xu XQ, Wu FY (2020). Utility of T2 mapping in the staging of thyroid-associated ophthalmopathy: efficiency of region of interest selection methods. Acta Radiol.

[30] He Y, Mu K, Liu R, Zhang J, Xiang N (2017). Comparison of two different regimens of intravenous methylprednisolone for patients with moderate to severe and active Graves' ophthalmopathy: a prospective, randomized controlled trial. Endocr J.

[31] Hou K, Ai T, Hu WK, Luo B, Wu YP, Liu R (2017). Three dimensional orbital magnetic resonance T2-mapping in the evaluation of patients with Graves' ophthalmopathy. J Huazhong Univ Sci Technolog Med Sci.

[32] Liu P, Luo B, Chen L, Wang QX, Yuan G, Jiang GH, Zhang J (2021). Baseline volumetric T2 relaxation time histogram analysis: can it be used to predict the response to intravenous methylprednisolone therapy in patients with thyroid-associated ophthalmopathy?. Front Endocrinol (Lausanne).

[33] Utech CI, Khatibnia U, Winter PF, Wulle KG (1995). MR T2 relaxation time for the assessment of retrobulbar inflammation in Graves' ophthalmopathy. Thyroid.

[34] Cohen LM, Liou VD, Cunnane ME, Yoon MK (2020) Radiographic analysis of fatty infiltration of the extraocular muscles in thyroid eye disease. Orbit: 1–6. 10.1080/01676830.2020.1817100 10.1080/01676830.2020.1817100 32878536

[35] McNab AA (2020). Orbital myositis: a comprehensive review and reclassification. Ophthalmic Plast Reconstr Surg.

[36] Savino G, Midena G, Tartaglione T, Milonia L, Caputo CG, Grimaldi G (2020). Clinical-radiological patterns and histopathological outcomes in non-thyroid extraocular muscle enlargement: retrospective case series and current concepts. Ophthalmic Plastic Reconstructive Surg.

[37] Mombaerts I, Koornneef L (1997). Current status in the treatment of orbital myositis. Ophthalmology.

[38] Mannor GE, Rose GE, Moseley IF, Wright JE 1997 Outcome of orbital myositis. Clinical features associated with recurrence. Ophthalmology 104: 409–413; discussion, 414. 10.1016/s0161-6420(97)30300-5 10.1016/s0161-6420(97)30300-5 9082264

[39] MS Kang HK Yang N Kim JM Hwang 2020 Clinical features of ocular motility in idiopathic orbital myositis J Clin Med 9. 10.3390/jcm9041165 10.3390/jcm9041165 PMC7231042 32325733

[40] Dresner SC, Rothfus WE, Slamovits TL (1984). Computed tomography of orbital myositis. American J Roentgenology.

[41] Jakobiec FA, Syed ZA, Stagner AM, Harris GJ, Rootman J, Yoon MK, Mombaerts I (2016). Orbital inflammation in pregnant women. Am J Ophthalmol.

[42] Ben Simon GJ, Syed HM, Douglas R, McCann JD, Goldberg RA (2004). Extraocular muscle enlargement with tendon involvement in thyroid-associated orbitopathy. Am J Ophthalmol.

[43] Yawar B, Malik Z, Naz F (2020). A rare case of orbital myositis. J Ayub Med Coll Abbottabad.

[44] Kang MS, Yang HK, Kim N, Hwang JM (2020) Clinical features of ocular motility in idiopathic orbital myositis. J Clin Med 9. 10.3390/jcm9041165 10.3390/jcm9041165 PMC7231042 32325733

[45] Weber AL, Romo LV, Sabates NR (1999). Pseudotumor of the orbit: clinical, pathologic, and radiologic evaluation. Radiologic Clin North America.

[46] Lacey B, Chang W, Rootman J (1999). Nonthyroid causes of extraocular muscle disease. Survey Ophthalmol.

[47] Douglas VP, Douglas KAA, Rizzo JF, Chwalisz BK (2020). Case report: Orbital myositis triggering oxygen-responsive cluster headache. Cephalalgia.

[48] Hattori H, Ohnishi S, Nakagawa Y, Ikemiya M, Yamato K, Matsuoka O, Yokoyama T, Yamano T (2005). An infant with idiopathic orbital myositis poorly responsive to steroid therapy: A case report. Brain and Development.

[49] Zheng Y, Zhang YX, Ding MP (2020). Treatment of idiopathic orbital myositis with frequent relapses: first case with tacrolimus and review of literature. J Neuroimmunol.

[50] Wu NA, Sun FY (2019). Clinical observation of orbital IgG4-related disease. Experiment Therapeutic Med.

[51] Wallace ZS, Khosroshahi A, Jakobiec FA, Deshpande V, Hatton MP, Ritter J, Ferry JA, Stone JH (2012). IgG4-related systemic disease as a cause of "idiopathic" orbital inflammation, including orbital myositis, and trigeminal nerve involvement. Surv Ophthalmol.

[52] Alsoudi A, Copperman TS, Idowu OO, Kersten RC (2019). Occult nasolacrimal duct obstruction secondary to IgG4-related ophthalmic disease. Ophthalmic Plastic Reconstructive Surger.

[53] Erdei A, Steiber Z, Molnar C, Berenyi E, Nagy EV (2018). Exophthalmos in a young woman with no graves' disease - a case report of IgG4-related orbitopathy. BMC Ophthalmol.

[54] Ginat DT, Freitag SK, Kieff D, Grove A, Fay A, Cunnane M, Moonis G (2013). Radiographic patterns of orbital involvement in igg4-related disease.. Ophthalmic Plastic Reconstructive Surg.

[55] Hardy TG, McNab AA, Rose GE (2014). Enlargement of the infraorbital nerve: an important sign associated with orbital reactive lymphoid hyperplasia or immunoglobulin G4-related disease.. Ophthalmology.

[56] Inaba H, Hayakawa T, Miyamoto W, Takeshima K, Yamaoka H, Furukawa Y, Kawashima H, Ariyasu H, Wakasaki H, Furuta H, Nishi M, Nakao T, Sasaki H, Okada Y, Matsunaga K, Nakamura Y, Akamizu T (2013). IgG4-related ocular adnexal disease mimicking thyroid-associated orbitopathy. Internal Med.

[57] Cheng S, Vu P (2009). Recurrent orbital myositis with radiological feature mimicking thyroid eye disease in a patient with Crohn's disease. Orbit.

[58] Ishihara R, Jain SF, Perry D, Reinhardt A, Suh D, Legge R (2020) Orbital pseudotumor as the presenting symptom of Crohn's disease in a male child. American J Ophthalmol Case Reports 18. 10.1016/j.ajoc.2020.100669 10.1016/j.ajoc.2020.100669 PMC7090333 32215344

[59] Ramalho J, Castillo M (2008). Imaging of orbital myositis in Crohn's disease. Clinical Imaging.

[60] Mehta D, Tabbaa M (2016). Rare ocular manifestation of IBD. American J Gastroenterol.

[61] Zenone T (2014). Orbital myositis and Crohn's disease. Inter J Rheumatic Diseases.

[62] Mavrikakis I, Rootman J (2007). Diverse clinical presentations of orbital sarcoid. Am J Ophthalmol.

[63] Malik R, Plant GT (1999). Sarcoid orbital myositis. Neuro-Ophthalmol.

[64] Campagna G, Prospero Ponce CM, Vickers A, Hong BYB, Pellegrini F, Cirone D, Romano F, Machin P, Lee AG 2020 Neuro-ophthalmic sarcoidosis. Neuro-Ophthalmol 44: 319–326. 10.1080/01658107.2019.1583761 10.1080/01658107.2019.1583761 PMC7518323 33012922

[65] Kim JS, Scawn RL, Lee BW, Lin JH, Korn BS, Kikkawa DO (2016). Masquerading orbital sarcoidosis with isolated extraocular muscle involvement. Open Ophthalmol J.

[66] Hayashi Y, Ishii Y, Nagasawa J, Arai S, Okada H, Ohmi F, Umetsu T, Machida Y, Kurasawa K, Takemasa A, Suzuki S, Senoh T, Sada T, Hirata K (2016). Subacute sarcoid myositis with ocular muscle involvement; a case report and review of the literature. Sarcoidosis Vasc Diffuse Lung Dis.

[67] Boddu N, Jumani M, Wadhwa V, Bajaj G, Faas F 2017 Not all orbitopathy is Graves': Discussion of cases and review of literature. Front Endocrinology 8. 10.3389/fendo.2017.00184 10.3389/fendo.2017.00184 PMC5534452 28824545

[68] Simon EM, Zoarski GH, Rothman MI, Numaguchi Y, Zagardo MT, Mathis JM (1998). Systemic sarcoidosis with bilateral orbital involvement: MR findings. Am J Neuroradiol.

[69] Ismailova DS, Abramova JV, Novikov PI, Grusha YO (2018). Clinical features of different orbital manifestations of granulomatosis with polyangiitis. Graefes Arch Clin Exp Ophthalmol.

[70] Bahrami B, Juniat V, Davis G, Selva D (2020). Pachymeningeal enhancement on magnetic resonance imaging in granulomatosis with polyangiitis.. Can J Ophthalmol.

[71] Franco J, Lee NG 2020 Granulomatosis with polyangiitis presenting as recurrent, multifocal orbital myositis. Orbit (London). 10.1080/01676830.2020.1817949 10.1080/01676830.2020.1817949 32878531

[72] Salam A, Meligonis G, Malhotra R (2008). Superior oblique myositis as an early feature of orbital Wegener's granulomatosis.. Orbit.

[73] Lefebvre DR, Reinshagen KL, Yoon MK, Stone JH, Stagner AM (2018). Case 39–2018: An 18-Year-Old Man with Diplopia and Proptosis of the Left Eye. N Engl J Med.

[74] Franco J, Lee NG 2020 Granulomatosis with polyangiitis presenting as recurrent, multifocal orbital myositis. Orbit: 1–3 10.1080/01676830.2020.1817949 10.1080/01676830.2020.1817949 32878531

[75] Savino G, Midena G, Tartaglione T, Milonia L, Caputo CG, Grimaldi G (2020). Clinical-radiological patterns and histopathological outcomes in non-thyroid extraocular muscle enlargement: retrospective case series and current concepts. Ophthalmic Plast Reconstr Surg.

[76] Tsironi E, Eftaxias B, Karabatsas CH, Ioachim E, Kalogeropoulos C, Psilas K (2005). An unusually longstanding, strictly ocular, limited form of Wegener's granulomatosis. Acta Ophthalmol Scand.

[77] Arrico L, Abbouda A, Bianchi S, Malagola R (2014). Acute monolateral proptosis and orbital myositis in a patient with discoid lupus erythematosus: a case report. J Med Case Rep.

[78] Kono S, Takashima H, Suzuki D, Terada T, Konishi T, Miyajima H (2014). Orbital myositis associated with discoid lupus erythematosus. Lupus.

[79] Selva D, Dolman PJ, Rootman J (2000). Orbital granulomatous giant cell myositis: a case report and review. Clin Experimental Ophthalmol.

[80] Mombaerts I, Bilyk JR, Rose GE, McNab AA, Fay A, Dolman PJ, Allen RC, Devoto MH, Harris GJ, Bernardini FP, Bonavolonta G, Codere F, Cockerham KP, Cruz AAV, Dutton JJ, Garrity JA, Goldberg RA, Grove AS, Kazim M, Kennerdell JS, Kersten RC, Kim YD, Levine MR, Lucarelli MJ, Mourits MP, Nerad JA, Orcutt JC, Perry JD, Putterman AM, Seah LL, Selva D, Sivak-Callcott JA, Stefanyszyn MA, Strianese D, Sullivan TJ, Verity D (2017). Consensus on diagnostic criteria of idiopathic orbital inflammation using a modified delphi approach. JAMA Ophthalmology.

[81] Klein BR, Hedges Iii TR, Dayal Y, Adelman LS (1989). Orbital myositis and giant cell myocarditis. Neurology.

[82] Jui-Chi Chang R, Kuang V, Meyer J, Chang E, Roberts-Thomson SJ, McKelvie P, Hardy TG, Pick ZS 2020 Orbital giant cell myositis is an unusual and potentially lethal cause of bilateral ophthalmoplegia - a case report and literature review. Orbit: 1–7. 10.1080/01676830.2020.1856144 10.1080/01676830.2020.1856144 33297808

[83] Caramaschi P, Biasi D, Carletto A, Bambara LM (2003). Orbital myositis in a rheumatoid arthritis patient during etanercept treatment. Clin Exp Rheumatol.

[84] Ssi-Yan-Kai I, Pearson A (2012). Orbital myositis and psoriatic arthritis. Can J Ophthalmol.

[85] Spitzer SG, Bersani TA, Mejico LJ (2005). Multiple bilateral extraocular muscle metastases as the initial manifestation of breast cancer. J neuro-ophthalmol : official j North American Neuro-Ophthalmol Soc.

[86] Shields JA, Shields CL, Scartozzi R (2004). Survey of 1264 patients with orbital tumors and simulating lesions: The 2002 Montgomery Lecture, part 1. Ophthalmology.

[87] Watkins LM, Carter KD, Nerad JA (2011). Ocular adnexal lymphoma of the extraocular muscles: case series from the University of Iowa and review of the literature. Ophthalmic plastic reconstructive surg.

[88] Behbehani RS, Bilyk JR, Haber MM, Savino PJ (2005). Orbital lymphoma with concomitant sarcoid-like granulomas. Ophthalmic Plast Reconstr Surg.

[89] Guo PD, Xian JF, Man FY, Liu ZH, Yan F, Zhao J, Wang ZC (2016). Magnetic resonance imaging features of extraocular muscle lymphoma in five cases. Chinese med j.

[90] Izambart C, Robert PY, Petellat F, Petit B, Gastaud P, Lagier J, Labrousse F, Adenis JP (2008). Extraocular muscle involvement in marginal zone B-cell lymphomas of the orbit. Orbit.

[91] Malik KJ, Berntson DG, Harrison AR (2006). Lymphoplasmacytic lymphoma isolated to an extraocular muscle. Ophthal plastic reconstructive surg.

[92] Payne JF, Shields CL, Eagle RC, Shields JA (2006). Orbital lymphoma simulating thyroid orbitopathy. Ophthal plastic reconstructive surg.

[93] Rossman D, Michel R, Codere F (2009). A case of an enlarged medial rectus muscle. Inter ophthalmol.

[94] Stenman L, Persson M, Enlund F, Clasen-Linde E, Stenman G, Heegaard S (2016). Primary orbital precursor t-cell lymphoblastic lymphoma: report of a unique case. Molecular and Clinical Oncology.

[95] Surov A, Behrmann C, Holzhausen HJ, Kösling S (2011). Lymphomas and metastases of the extra-ocular musculature. Neuroradiology.

[96] Truong T, Char DH, Dillon WP (1988). Superior oblique muscle neoplasms. Orbit.

[97] Shafi F, Mathewson P, Mehta P, Ahluwalia HS (2017). The enlarged extraocular muscle: to relax, reflect or refer?. Eye (Lond).

[98] Polito E, Galieni P, Leccisotti A (1997). Bilateral optic nerve compression caused by lymphomatous infiltration of all extraocular muscles. Neuro-Ophthalmology.

[99] Kawakami H, Mochizuki K, Goto H, Watanabe N, Tanaka T 2017 Orbital T-cell lymphoma with discrete enlargements of all extraocular muscles bilaterally in patient with moon face countenance. Case reports in ophthalmological medicine 2017: 8902162. 10.1155/2017/8902162 10.1155/2017/8902162 PMC5401722 28487798

[100] Jung JH, Oh EH, Shin DH, Choi SY, Choi KD, Choi JH (2019). Orbital lymphoma presenting with inferior rectus palsy. J Clin Neurol (Korea).

[101] Ejstrup R, Mikkelsen LH, Andersen MK, Clasen-Linde E, Gjerdrum LMR, Safavi S, Heegaard S (2019). Orbital precursor B-lymphoblastic lymphoma involving the extraocular muscles in a 56-year-old male and a review of the literature. Oncology Lett.

[102] Hornblass A, Jakobiec FA, Reifler DM, Mines J (1987). Orbital lymphoid tumors located predominantly within extraocular muscles. Ophthalmology.

[103] Lacey B, Chang W, Rootman J (1999) Nonthyroid causes of extraocular muscle disease. Surv Ophthalmol 44: 187–213 10.1016/s0039-6257(99)00101-0 10.1016/s0039-6257(99)00101-0 10588439

[104] Framarino-Dei-Malatesta M, Chiarito A, Bianciardi F, Fiorelli M, Ligato A, Naso G, Pecorella I (2019). Metastases to extraocular muscles from breast cancer: case report and up-to-date review of the literature. BMC cancer.

[105] Wang Y, Mettu P, Maltry A, Harrison A, Mokhtarzadeh A (2017) Metastatic breast carcinoma to the superior oblique in a male. Ophthalmology and Therapy 6: 355–359. 10.1007/s40123-017-0093-7 10.1007/s40123-017-0093-7 PMC5693831 28550385

[106] Choi JH, Park HJ, Choi KG, Lim KH, Park KD 2017 Unilateral ptosis with bilateral incomplete ophthalmoplegia as the initial presentation in metastatic cancer. EWHA Medical Journal 40: 136-139. 10.12771/emj.2017.40.3.136

[107] Luneau K, Falardeau J, Hardy I, Boulos PR, Boghen D (2007). Ophthalmoplegia and lid retraction with normal initial orbit CT imaging in extraocular muscle metastases as the presenting sign of breast carcinoma. Journal neuro-ophthalmol : official j North American Neuro-Ophthalmol Soc.

[108] Milman T, Pliner L, Langer PD (2008). Breast carcinoma metastatic to the orbit: an unusually late presentation. Ophthalmic plastic reconstructive surg.

[109] Peckham EL, Giblen G, Kim AK, Sirdofsky MD (2005). Bilateral extraocular muscle metastasis from primary breast cancer. Neurology.

[110] Polito E, Leccisotti A (1993). Extraocular muscle enlargement as an isolated finding. A CT and MRI differential diagnosis. Orbit.

[111] Wiggins RE, Byrne SF (2012). Metastatic tumor to the extraocular muscles: report of 5 cases. J AAPOS : official publ American Association Pediatric Ophthalmol Strabismus.

[112] Yabaş Kızıloğlu Ö, Paksoy Türköz F, Totuk Gedar ÖM, Mestanoğlu M, Yapıcıer Ö (2019). Breast Carcinoma Metastasis to the Medial Rectus Muscle: Case Report. Turkish j ophthalmol.

[113] Coutinho I, Marques M, Almeida R, Custódio S, Silva TS, Águas F (2018). Extraocular muscles involvement as the initial presentation in metastatic breast cancer. J Breast Cancer.

[114] Salinas-Botrán A, Guarín-Corredor MJ (2019). Orbital Metastasis in Breast Cancer. The New England j med.

[115] Greene DP, Shield DR, Shields CL, Shields JA, Servat JJ, Lin CJ, Douglass AM, Fulco EA, Levin F (2014). Cutaneous melanoma metastatic to the orbit: review of 15 cases. Ophthal plastic reconstructive surg.

[116] Krema H, Dawson LA, Hogg D, Laperriere N (2013). Bilateral extraocular muscles metastases from a choroidal melanoma. Canadian j ophthalmol J canadien d'ophtalmologie.

[117] Capone A, Jr Slamovits TL 1990 Discrete metastasis of solid tumors to extraocular muscles. Archives of ophthalmology (Chicago, Ill : 1960) 108: 237–243. 10.1001/archopht.1990.01070040089037 10.1001/archopht.1990.01070040089037 2405828

[118] Shih CY, Mirchandani G, Kazim M (2007). Atypical MRI features of intraorbital metastatic melanoma. Ophthal plastic reconstructive surg.

[119] Pirlamarla AK, Tang J, Amin B, Kabarriti R (2018). Vulvar melanoma with isolated metastasis to the extraocular muscles: case report and brief literature review. Anticancer Res.

[120] Gupta A, Chazen JL, Phillips CD (2011). Carcinoid tumor metastases to the extraocular muscles: MR imaging and CT findings and review of the literature. AJNR Am J Neuroradiol.

[121] Kamieniarz L, Armeni E, O'Mahony LF, Leigh C, Miah L, Narayan A, Bhatt A, Cox N, Mandair D, Navalkissoor S, Caplin M, Toumpanakis C (2020). Orbital metastases from neuroendocrine neoplasms: clinical implications and outcomes. Endocrine.

[122] Ryan TG, Juniat V, Stewart C, Malhotra R, Hardy TG, McNab AA, Davis G, Selva D 2021 Clinico-radiological findings of neuroendocrine tumour metastases to the orbit. Orbit: 1–9. 10.1080/01676830.2021.1895845 10.1080/01676830.2021.1895845 33729098

[123] Bohman E, Sahlin S, Seregard S (2009). Carcinoid presenting as extrinsic eye muscle metastasis. Acta Ophthalmol.

[124] Khaw P, Ball D, Duchesne G (2001). Carcinoid tumour of the orbital muscles: a rare occurrence. Australasian radiol.

[125] Kiratli H, Yilmaz PT, Yildiz ZI (2008). Metastatic atypical carcinoid tumor of the inferior rectus muscle. Ophthal plastic reconstructive surg.

[126] Matsuo T, Ichimura K, Tanaka T, Takenaka T, Nakayama T (2010) Neuroendocrine tumor (carcinoid) metastatic to orbital extraocular muscle: case report and literature review. Strabismus 18: 123-128. 10.3109/09273972.2010.525779 10.3109/09273972.2010.525779 21091332

[127] Borota OC, Kloster R, Lindal S (2005). Carcinoid tumour metastatic to the orbit with infiltration to the extraocular orbital muscle. APMIS : acta pathologica, microbiologica, et immunologica Scandinavica.

[128] Hatsis AJ, Henry RK, Curtis MT, Bilyk JR, Sivalingam MD, Eagle RC, Milman T 2020 Ocular adnexal manifestations of neuroendocrine neoplasms: a case report and a major review. Orbit: 1–11. 10.1080/01676830.2020.1839108 10.1080/01676830.2020.1839108 33140682

[129] Sivagnanavel V, Riordan-Eva P, Jarosz J, Portmann B, Buxton-Thomas M (2004). Bilateral orbital metastases from a neuroendocrine tumor. J Neuro-ophthalmol : Official J North American Neuro-Ophthalmol Soc.

[130] Kozubowska K, Skorek A, Pęksa R (2019). Neuroendocrine tumour metastasis to the orbit. Endokrynologia Polska.

[131] Lekse JM, Zhang J, Mawn LA (2003). Metastatic gastroesophageal junction adenocarcinoma to the extraocular muscles. Ophthalmology.

[132] Souayah N, Krivitskaya N, Lee HJ (2008). Lateral rectus muscle metastasis as the initial manifestation of gastric cancer. J Neuro-ophthalmol : official J North American Neuro-Ophthalmol Soc.

[133] Goto S, Takeda H, Sasahara Y, Takanashi I, Yamashita H (2019). Metastasis of advanced gastric cancer to the extraocular muscle: a case report. J Med Case Rep.

[134] Julve M, Maviki M, Gillmore R 2014 Bilateral extraocular muscle (EOM) metastases from adenocarcinoma of the gastro-oesophageal junction (GOJ). BMJ case reports 2014. 10.1136/bcr-2014-205368 10.1136/bcr-2014-205368 PMC4139542 25115784

[135] Dodd MU, Wolkow N, Cunnane ME, Ma L, Dryja TP, Hunter D (2020). Isolated orbital amyloidosis causing internal and external ophthalmoplegia. J aapos.

[136] Paula JS, Paula SA, Cruz AA, Chahud F (2008). Superior oblique muscle amyloidosis mimicking myositis. Ophthalmic Plast Reconstr Surg.

[137] Katz B, Leja S, Melles RB, Press GA (1989). Amyloid ophthalmoplegia. Ophthalmoparesis secondary to primary systemic amyloidosis. J Clin Neuroophthalmol.

[138] Tong JY, Juniat V, McKelvie PA, O'Donnell BA, Hardy TG, McNab AA, Selva D 2021 Clinical and radiological features of intramuscular orbital amyloidosis: a case series and literature review. Ophthalmic Plast Reconstr Surg. 10.1097/iop.0000000000002061 10.1097/IOP.0000000000002061 34516528

[139] Okamoto K, Ito J, Emura I, Kawasaki T, Furusawa T, Sakai K, Tokiguchi S (1998). Focal orbital amyloidosis presenting as rectus muscle enlargement: CT and MR findings. AJNR Am J Neuroradiol.

[140] Nishikawa N, Kawaguchi Y, Konno A, Kitani Y, Takei H, Yanagi Y (2021). Primary isolated amyloidosis in the extraocular muscle as a rare cause of ophthalmoplegia: a case report and literature review. Am J Ophthalmol Case Rep.

[141] Shah VS, Cavuoto KM, Capo H, Grace SF, Dubovy SR, Schatz NJ (2016). Systemic amyloidosis and extraocular muscle deposition. J Neuroophthalmol.

[142] Monteiro ML, Gonçalves AC, Bezerra AM (2016). Isolated primary amyloidosis of the inferior rectus muscle mimicking Graves' orbitopathy. Einstein (Sao Paulo).

[143] Li Y, Wang Y, Zhang W (2020). A case of isolated amyloidosis in extraocular muscle mimicking thyroid eye disease. J aapos.

[144] Murdoch IE, Sullivan TJ, Moseley I, Hawkins PN, Pepys MB, Tan SY, Garner A, Wright JE (1996). Primary localised amyloidosis of the orbit. Br J Ophthalmol.

[145] Chakrabortty S, Nagashima T, Izawa I, Sekiya Y, Sugiura T, Inoue M, Imai Y, Ehara K, Tamaki N (1993). Intraorbital arteriovenous malformation: case report. Surg Neurol.

[146] Kamboj A, Tooley AA, Godfrey KJ, Maher MD, Schubert HD, Kazim M (2020) Extraocular muscle enlargement in retinoencephalofacial angiomatosis. Orbit 39: 221-223. 10.1080/01676830.2019.1677727 10.1080/01676830.2019.1677727 31658870

[147] Patrinely JR, Osborn AG, Anderson RL, Whiting AS (1989). Computed tomographic features of nonthyroid extraocular muscle enlargement. Ophthalmology.

[148] Ahmadi J, Teal JS, Segall HD, Zee CS, Han JS, Becker TS (1983). Computed tomography of carotid-cavernous fistula. AJNR Am J Neuroradiol.

[149] Kim D, Choi YJ, Song Y, Chung SR, Baek JH, Lee JH (2020). Thin-section MR imaging for carotid cavernous fistula. American Journal of Neuroradiology.

[150] Komiyama M, Fu Y, Yagura H, Yasui T, Hakuba A, Nishimura S (1990). MR imaging of dural AV fistulas at the cavernous sinus. J Comput Assist Tomogr.

[151] Chan HHL, Hardy TG, McNab AA (2019). Spontaneous orbital hemorrhage related to the extraocular muscles. Ophthalmic Plast Reconstr Surg.

[152] Ben Simon GJ, McNab AA (2005). Idiopathic orbital hemorrhage related to the inferior rectus muscle: a rare cause for acute-onset diplopia and unilateral proptosis. Ophthalmology.

[153] Landau Prat D, Greenberg G, McNab AA, Ben Simon G, Ben Simon G, Greenberg G, Landau Prat D (2022). Nontraumatic Orbital Hemorrhage (NTOH). Atlas of Orbital Imaging.

[154] Hakin KN, McNab AA, Sullivan TJ (1994). Spontaneous hemorrhage within the rectus muscle. Ophthalmology.

[155] Sundaram PM, Jayakumar N, Noronha V (2004). Extraocular muscle cysticercosis - a clinical challenge to the ophthalmologists. Orbit.

[156] Rath S, Honavar SG, Naik M, Anand R, Agarwal B, Krishnaiah S, Sekhar GC (2010). Orbital cysticercosis clinical manifestations, diagnosis, management, and outcome. Ophthalmology.

[157] Sekhar GC, Honavar SG (1999). Myocysticercosis: experience with imaging and therapy. Ophthalmology.

[158] Ding J, Zhao H, Lin J (2015). Surgical excision of orbital cysticercosis lodged in superior oblique muscle: clinical case report. Medicine (Baltimore).

[159] Shashni A, Pujari A, Bajaj MS, Kumar P (2018). Superior oblique muscle cysticercosis: importance of long-term assessment by a single observer. Can J Ophthalmol.

[160] Labh RK, Sharma AK (2013). Ptosis: a rare presentation of ocular cysticercosis. Nepal J Ophthalmol.

[161] Chopra R, Kapoor H, Chopra A (2012). Ocular myocysticercosis: favorable outcomes with early diagnosis and appropriate therapy. Nepal J Ophthalmol.

[162] Agrawal S, Ranjan S, Mishra A (2013) Ocular myocysticercosis: an unusual case of ptosis. Nepal J Ophthalmol 5: 279–281. 10.3126/nepjoph.v5i2.8745 10.3126/nepjoph.v5i2.8745 24172571

[163] Saravi SS, Sabouni F, Arefidoust A, Yaftian R, Heydarzadeh S, Rajabi MT (2016) An orbital hydatid cyst involving inferior rectus muscle: a case report. Orbit 35: 109-112. 10.3109/01676830.2015.1099705 10.3109/01676830.2015.1099705 26905024

[164] Acharya IG, Jethani J (2010). Pyomyositis of extraocular muscle: case series and review of the literature. Indian J Ophthalmol.

[165] Chandraparnik P, Lumyongsatien M, Selva D (2021) Multifocal extraocular muscle pyomyositis: A case report and review of literature. Orbit 40: 258-262. 10.1080/01676830.2020.1768558 10.1080/01676830.2020.1768558 32515624

[166] Varma A, Sharma K, Rathi B, Gupta RK, Malik V (2003). Isolated abscess of extraocular muscle in two young boys: clinical and imaging features. Orbit.

[167] Haufschild T, Weber P, Nuttli I, Hecker B, Flammer J, Kaiser HJ (2004). Idiopathic isolated abscess in an extraocular muscle in a child. Arch Ophthalmol.

[168] Agius MdB, Vella M (2015). A rare case of an idiopathic extraocular muscle abscess. Malta Med J.

[169] Mombaerts I, Rose GE, Verity DH (2017). Diagnosis of enlarged extraocular muscles: when and how to biopsy. Curr Opin Ophthalmol.

[170] Jyani R, Ranade D, Joshi P (2020). Spectrum of orbital cellulitis on magnetic resonance imaging. Cureus.

[171] Tsirouki T, Dastiridou AI, Ibánez flores N, Cerpa JC, Moschos MM, Brazitikos P, Androudi S (2018). Orbital cellulitis. Surv Ophthalmol.

